# Biodiesel production potential from fat fraction of municipal waste in Makkah

**DOI:** 10.1371/journal.pone.0171297

**Published:** 2017-02-16

**Authors:** K. Shahzad, A. S. Nizami, M. Sagir, M. Rehan, S. Maier, M. Z. Khan, O. K. M. Ouda, I. M. I. Ismail, A. O. BaFail

**Affiliations:** 1 Center of Excellence in Environmental Studies (CEES), King Abdulaziz University, Jeddah, Saudi Arabia; 2 Chemical Engineering Department, University of Gujrat, Gujrat, Pakistan; 3 Institute for Process and Particle Engineering, Graz University of Technology, Graz, Austria; 4 Environmental Research Laboratory, Department of Chemistry, Aligarh Muslim University, Aligarh, Uttar Pradesh, India; 5 Department of Civil Engineering, Prince Mohamed Bin Fahd University, Al Khobar, Saudi Arabia; 6 Department of Industrial Engineering, King Abdulaziz University, Jeddah, Saudi Arabia; Leibniz-Institut fur Pflanzengenetik und Kulturpflanzenforschung Gatersleben, GERMANY

## Abstract

In the Kingdom of Saudi Arabia (KSA), millions of Muslims come to perform Pilgrimage every year. Around one million ton of municipal solid waste (MSW) is generated in Makkah city annually. The collected MSW is disposed of in the landfills without any treatment or energy recovery. As a result, greenhouse gas (GHG) emissions and contamination of the soil and water bodies along with leachate and odors are occurring in waste disposal vicinities. The composition of MSW shows that food waste is the largest waste stream (up to 51%) of the total generated MSW. About 13% of the food waste consists of fat content that is equivalent to about 64 thousand tons per year. This study aims to estimate the production potential of biodiesel first time in Makkah city from fat/oil fractions of MSW and highlight its economic and environmental benefits. It has been estimated that 62.53, 117.15 and 6.38 thousand tons of biodiesel, meat and bone meal (MBM) and glycerol respectively could be produced in 2014. A total electricity potential of 852 Gigawatt hour (GWh) from all three sources based on their energy contents, Higher Heating Value (HHV) of 40.17, 18.33 and 19 MJ/kg, was estimated for 2014 that will increase up to 1777 GWh in 2050. The cumulative net savings from landfill waste diversion (256 to 533 million Saudi Riyal (SAR)), carbon credits (46 to 96 million SAR), fuel savings (146 to 303 million SAR) and electricity generation (273 to 569 million SAR) have a potential to add a total net revenue of 611 to 1274 million SAR every year to the Saudi economy, from 2014 to 2050 respectively. However, further studies including real-time data about annual slaughtering activities and the amount of waste generation and its management are critical to decide optimum waste management practices based on life cycle assessment (LCA) and life cycle costing (LCC) methodologies.

## 1. Introduction

The increased cost of raw materials production and the competition among economic sectors has encouraged the researchers and policy makers to investigate the cost effective measures for utilizing untapped material resources through the cleaner production technologies [1]. One of the cheap untapped resources is the use of waste streams as feedstocks for energy and value-added products (VAP) recovery through various techniques, which will not only reduce the process cost but also minimize the waste-related environmental issues [2–3]. The sustainable management of municipal solid waste (MSW) is a chronic environmental and strategic problem in most developing nations, including the Kingdom of Saudi Arabia (KSA) [4–5].

KSA has one of the largest tourist industry in the world due to the presence of two holiest places for Muslims; Masjid-ul-Haram in Makkah and Masjid-e-Nabwi in Medina [6]. Millions of Muslims from all over the world visit these places every year to perform Pilgrimage [7]. The Pilgrim number is significantly increasing every year due to continuous improvements in transportation, accommodation, availability of food supply and better security services. As a result, MSW generation in Makkah city has increased significantly (up to 1 million ton per year) in recent years [8]. Most of the collected MSW is disposed to the landfills without any treatment or material and energy recovery, as no material recovery facility (MRF) and waste-to-energy (WTE), plant exists [9,10].

Makkah city receives on average 2.4 thousand tons of MSW every day that reaches up to 3 thousand tons per day and 4.6 thousand tons per day during the months of Ramadan (month of fasting) and Hajj (Pilgrimage) respectively [2, 8]. The highest amount of waste is generated during last 10 days of Ramadan and 8–13 of Zulhijjah; the Pilgrimage time [7]. The total estimated amount of MSW generated in Makkah city during 2014 was 970 thousand tons, of which 880 thousand tons was generated by the local population, while 90 thousand tons was generated by Pilgrims [2]. The major waste constituents of MSW in Makkah city are food (50.6%), paper and cardboard (18.6%), and plastic wastes (17.4%) [2, 9].

**Fig 1:**
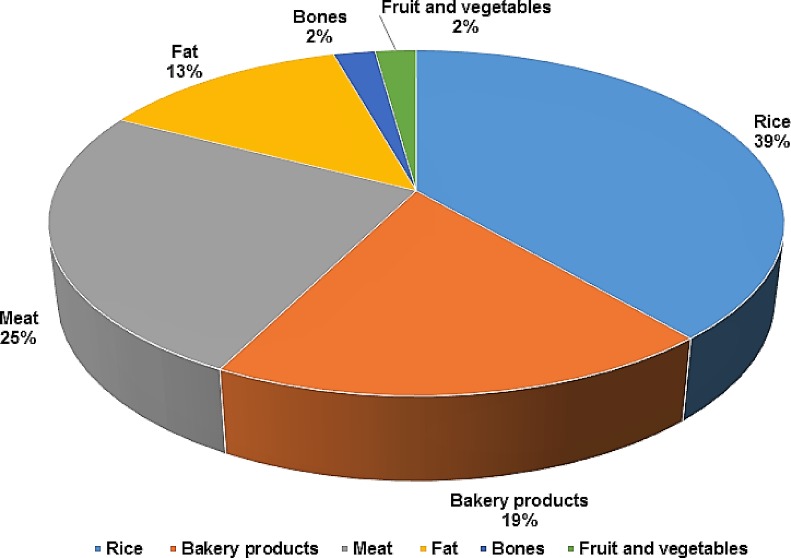
Distribution of food waste fractions in Makkah city [14].

The organic fractions of MSW, including food and slaughterhouse waste, and UCO are the main sources of MSW in Makkah city, especially during the Ramadan and Hajj seasons [6, 8]. In 2007’s Hajj period, around 700 thousand goats were slaughtered [7], while this number has increased almost four times (2.5 million animals) in 2014’s Hajj time [10]. The slaughterhouse waste consists of unwanted fractions of animals, including inedible body parts as well as blood and other by-products. These fractions can be up to 45% or more of an animal body weight depending on the use of different body parts [11]. The inedible parts can include feathers, blood vessels, ligaments, integuments and offal materials [12]. All of the animal’s related waste is disposed of untreated in Makkah. Similarly, food waste is the largest fraction of MSW (up to 50.6%) that is equivalent to 490 thousand ton every year. The physical composition of food waste (Fig 1) shows that it is the most suitable feedstock for a variety of WTE technologies such as anaerobic digestion (AD), composting and transesterification [13]. Further characterisation of MSW reveals that 13% of the annual food waste consisted of fat contents [14], which is equivalent to about 64 thousand ton of fat contents, including fats from edible fats and used cooking oils (UCOs) [2].

The Saudi Government is seeking sustainable solutions for Makkah city’s MSW management, especially for slaughterhouse waste in order to make this holiest city green and the event of Hajj, a ‘Green Hajj’ with least environmental pollution and maximum waste recycling [5, 7]. Therefore, first time, this study aims to estimate the biofuel production potential from fat/oil fraction of the MSW in the city of Makkah. The economic and ecological feasibility of biofuel production and ultimately the electricity production along with a reduction in greenhouse gas (GHG) emissions was carried. The paper is a worthy contribution as it sheds light on the significant waste generation problems in Makkah city, where millions of Pilgrims visit every year for religious rituals.

### 1.1 Process design for biodiesel production

**Fig 2:**
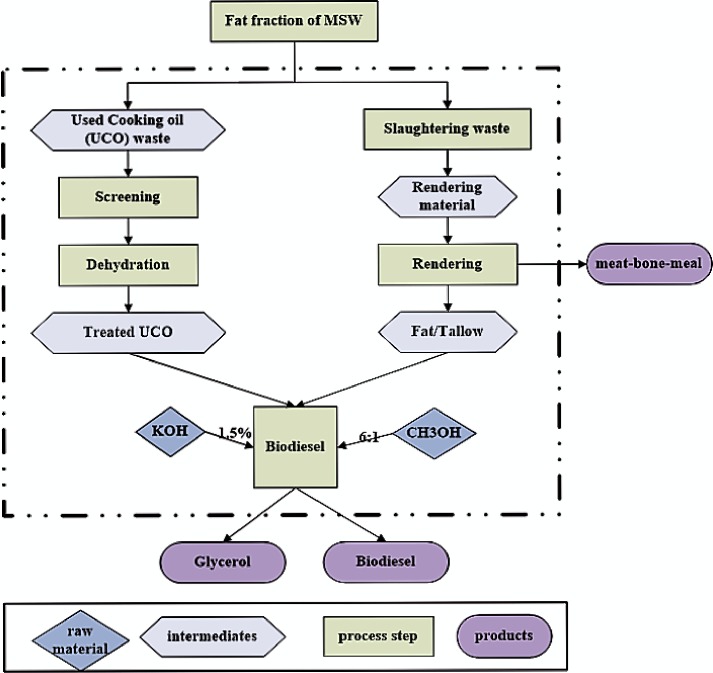
Process flow diagram for biodiesel production from fat/oil fraction of MSW in Makkah city.

According to process flow diagram (Fig 2), two main waste streams were contributing to the fat fraction of MSW, including UCO waste from the restaurants and slaughterhouse waste. The UCO consisted of waste vegetable oil as left over from homes and restaurant fryers. After collection and transportation to the biodiesel production facility, the first step is the screening of larger food particles and removal of related impurities such as water contents from the oil, as they prevent the transesterification reactions [15]. The waste generated from slaughterhouses is transported to the rendering facility. The slaughterhouse waste processed in the rendering facility mainly consists of fat, blood and bones. In the rendering process, the animal waste material is treated at 133°C for 20 minutes at 3 bar pressure to obtain tallow and protein-rich by-product such as meat and bone meal (MBM) [16]. Tallow can be used for several purposes, including use in the production of edible items, saponification process to make soaps, burning to fulfill energy requirements and as a raw material for biofuel production. Similarly, MBM is generally used as a fodder for livestock industry as well as burnt to obtain the useful energy.

The monoalkyl esters of fatty acids are known as biodiesel. They are produced by reacting triglycerides with monoalkyl alcohols (methyl or ethyl) through transesterification reactions in the presence of acid or base catalysts [17,18]. The selection of the catalyst depends on the amount of free fatty acids (FFAs) in the feedstock. The reported values of FFAs in rendered tallow are 15% to 20%, while in UCO these values are lower than 15% [19]. The feedstocks containing these high values of FFAs are difficult to handle in the esterification process. Although the alkane catalyzed transesterification process is faster that completes in 30 minutes as compared to acid catalysis, which requires 1–8 h for reaction completion than the acid-catalysed process, it is not feasible to treat feedstocks with high FFAs content. The alkaline catalyst reacts with FFAs and forms soap and water through a hydrolysis reaction. The water produced in this step further reacts with methyl ester to produce more FFAs that results in more soap through saponification process [20]. In the case of high FFAs content feedstock, a single-step transesterification can be carried out by using acid based catalyst. The reaction has a high yield of biodiesel production, however, the rate of reaction is relatively slower, which means the reactor have to withstand corrosive acidic environment for a longer period of time. The best solution to handle feedstock with high FFAs content is to apply two-step transesterification process. In the first-step or pre-treatment, the FFAs are converted into biodiesel by the application of acid catalyst through esterification. The remaining triglycerides in the feedstock are converted into biodiesel through transesterification by using alkaline based catalyst [21].

Biodiesel production is a well-developed state-of-the-art process that has high production efficiency (up to 98%) and 10% glycerol production as a by-product, with reference to fatty acids input [16]. The animal fat based biodiesel is known as tallow methyl ester (TME). In the first step sulphuric acid (H_2_SO_4_) is used as a catalyst to convert FFA into biodiesel through esterification process. In the 2 step tallow is reacted with methanol (CH_3_OH) in 1:6 ratio, in the presence of potassium hydroxide (KOH) as a catalyst [22]. The raw material consists of saturated and unsaturated fatty acids and consequently biodiesel quality depends on the amount of unsaturated fatty acids fraction. Higher the amount of unsaturated portion of fatty acids, higher is the quality of biodiesel. In the case of the higher amount of saturated fraction, biodiesel has lower quality and can cause knocking or plug flow problems in engines [23].

## 2. Methodology

The base year in this study was considered to be 2014, this is because the most recent available data on MSW amounts and its characterization including slaughterhouse waste from the year 2014 has been used for all the calculations. It has been calculated that 970 thousand tons of MSW was generated during 2014. The major contributor was food waste with 490 thousand tons. In accordance with the reported 13% fat contents of food waste in the city of Makkah [8], the estimated organic fat fraction was 63.8 thousand tons. The main sources of fat fractions were slaughterhouse waste and UCO. The estimations were made using 3.4% annual population growth rate and 1.19% annual increase in Pilgrims’ flux [8]. The waste estimation was based on waste generation rate of 1.4 kg per capita per day for local population for 365 days. The waste generation for Pilgrims was estimated using 1.9 kg per capita per day for 40 days, while for Ramadan 2/3 value of waste generated during Hajj days was used [2]. In 2014, the local population of Makkah was 1.73 million, while 2.06 million people came to perform religious rituals.

### 2.1 Estimations of UCOs and fats

There was no real information available on UCO waste in Makkah city, as a result of restaurants and household activities. It has been estimated that during 2013, about 5.77 million litres of UCO was produced in KSA [24], which was equivalent to 0.19 litre UCO/capita, based on the population of KSA in 2013. This per capita value was used to calculate the total amount of UCO generated in Makkah city, considering 1.73 million population of Makkah city and 2.06 million Pilgrims during 2014. Therefore, the calculated annual amount of UCO in Makkah city was 0.73 million litres or 728 tons. The slaughterhouse waste was considered as a source of the remaining fat fraction of MSW that was 63.1 thousand tons. The rendering technology was used to convert slaughterhouse waste into VAP such as lard, tallow, and MBM. The technology processes all the waste materials coming from the slaughterhouse in the form of offal, bones, and fatty tissues, remnants of dead animals, and meat processors by heating and drying simultaneously to separate fats and proteins in the form of tallow and MBM [25]. The amount of rendering material to be processed for obtaining an equivalent amount of fat content was calculated by using reverse calculations.

The scientific literature reveals that about 14% tallow and 24% MBM were produced from processing of rendering raw materials [22]. The estimated amounts of rendering raw materials and MBM based on tallow contents are shown in (Table 1). It has been reported that animal fat/tallow was generated at a rate of 14% of rendering waste [16], which was equivalent to 451 thousand tons of slaughterhouse waste. The rendering process produces tallow and protein-rich by-product known as MBM. The processing of 451 thousand tons of rendering waste to produce 63 thousand tons of tallow also produced 117 thousand tons of MBM at a production yield of 26%, in relation to rendering input. The electricity production potential from MBM has been estimated using its HHV value that was 18.33 MJ/kg [26].

**Table 1:** Projected values of different fat related fraction in Makkah from 2014 to 2050.

Year	Fat Fraction (thousand ton/yr)	Tallow (thousand ton/yr)	UCO (thousand ton/yr)	Rendering material (thousand ton/yr)	MBM (thousand ton/yr)
2014	63.81	63.08	0.73	450.56	117.15
2020	74.27	73.46	0.82	524.69	136.42
2026	85.09	84.01	1.08	600.08	156.02
2032	96.30	95.13	1.18	679.47	176.66
2038	107.98	106.70	1.28	762.13	198.15
2044	120.19	118.80	1.39	848.56	220.63
2050	133.02	131.51	1.51	939.34	244.23

The production potential of biodiesel as a main product and glycerol as a by-product was estimated using 98% and 10% production yields with reference to input quantity of raw materials such as UCO and tallow [16]. In this study, glycerol was also considered as an alternate source of energy to generate electricity. The high heating value (HHV) of biodiesel and glycerol were 40.17 MJ/kg and 19 MJ/kg respectively [27]. The HHV values for biodiesel, glycerol and MBM were used to estimate electricity potential of the overall process using 35% electricity generation efficiency (Table 2). The revenue generation was calculated based on electricity production from biofuel and VAP. The current electricity selling price of 0.32 SAR/KWh in KSA [28] was used to estimate the expected money flow.

**Table 2:** Electricity production potential from biodiesel, glycerol, and MBM.

Years	MBM Production Potential (thousand tons/yr)	Electricity potential from MBM (GWh/yr)	Biodiesel Production (thousand tons)	Electricity potential from biodiesel (GWh/yr)	Glycerol production (thousand tons/yr)	Electricity potential from glycerol (GWh/yr)	Total electricity production potential (GWh/yr)
2014	117.15	596.30	62.53	244.19	6.38	11.79	852.28
2020	136.42	694.41	72.79	284.25	7.43	13.72	992.38
2026	156.02	794.19	83.39	325.64	8.51	15.72	1135.55
2032	176.66	899.26	94.38	368.57	9.63	17.79	1285.61
2038	198.15	1008.65	105.82	413.26	10.80	19.95	1441.86
2044	220.63	1123.05	117.79	459.99	12.02	22.20	1605.24
2050	244.23	1243.19	130.36	509.07	13.30	24.57	1776.83

### 2.2 Estimations of environmental savings

The environmental savings were estimated using the method proposed by Intergovernmental Panel on Climate Change (IPCC) for methane (CH_4_) emission approximation from the waste fraction [29–31]. The particular method is given as:

$$Q=\left(MSW_{T}\times MSW_{F}\times MCF\times DOC\times DOC_{F}\times F\times \frac{16}{12}\;R\right)\left(1-OX\right)$$ (1)

The elaboration of used parameters is given as:

Q Total CH_4_ emission potential (ton/yr)
MSWT Total MSW fraction (ton/yr)
MSWF MSW Fraction disposed to the landfill.
MCF Methane Correction Factor
DOC Degradable organic carbon fraction.
DOCF Dissimilated organic fraction
F CH_4_ fraction in the landfill gas
R Methane recovery (ton/yr)
16/12 Molecular weight ratio between methane and carbon.
OX Oxidation factor.

In accordance with the fact that 100% MSW generated in Makkah city is disposed of in the landfills, the value of MSWF was set as 1. The default value of 0.6, for uncategorized MSW disposal sites (MSWDS), was used for MCF. Similarly, food waste fraction has a default value of 0.15 for DOC. Considering the incomplete biodegradation of DOC in the landfills, the default value of 0.77 was used for DOCF. The substrate under consideration contains high concentrations of fat and protein fractions, so it is assumed that landfill gas (LFG) contains 60% CH_4_ content, hence the value of F was 0.60. At the moment, there is no CH_4_ recovery from the landfill sites in Makkah city, so R and oxidation factor values were assumed zero. Putting these values along with projected cumulative values of slaughterhouse and UCO waste generation in Eq 1, provided the projections of CH_4_ emission profile, as shown in Table 3. The global warming potential (GWP) of CH_4_ in CO_2_ equivalents was calculated by using GWP value of 25 for CH_4_ over 100 years of time span [29]. At the moment, selling and buying of carbon credits are not in practice in KSA. It reflects the potential revenue generation in case carbon crediting system is implemented in KSA. The carbon credits value was calculated using US$ 23.20/ton CO_2_ equivalent and was considered as GHG emission savings [32].

**Table 3:** Cumulative projection of slaughterhouse waste and UCO with their relevant GHG emissions potential.

Years	Slaughterhouse waste + UCO (thousand tons)	CH_4_ emission potential (thousand tons)	GWP (thousand ton CO_2_ eq.)
2014	451.29	21.41	535.14
2020	525.51	24.93	623.15
2026	601.16	28.51	712.86
2032	680.65	32.28	807.11
2038	763.41	36.21	905.25
2044	849.95	40.32	1007.88
2050	940.85	44.63	1115.66

^a^ using IPCC methodology

^b^ based on GWP of 25 for methane (CH_4_).

### 2.3 Estimations of oil and gas savings

In KSA, almost 100% energy demand is fulfilled by fossil fuel burning. About 55% of country’s energy demand is covered by burning of crude oil, while rest of the 45% is met by burning the natural gas [33]. The estimated amount of fuel saving was calculated by using typical power plant heating-rate values. The heating rates for oil and gas-fed power plants are 10334 Btu/KWh and 10354 Btu/KWh respectively [34]. It was assumed that GHG emissions for an equivalent amount of energy generation from the alternate power generation process were equal to GHG emissions for energy production from crude oil and natural gas. The revenue generation from equal amounts of oil and gas savings were calculated using fuel prices of US$38.33/barrel (bbl.) of Brent crude oil and US$1.8/ million Btu (MMBtu) or US$1.85/ million cubic feet (Mcf) of natural gas [35].

### 2.4 Revenue calculation

The gross revenue generation by the facility consists of the sum of gate fee saving from landfill diversion, carbon credits, and revenue potential from an equivalent amount of fuel savings and electricity production from biodiesel, glycerol, and MBM. The revenue from electricity production potential, carbon credits and equivalent oil and gas savings was calculated as mentioned in sections 2.1, 2.2 and 2.3 respectively. Following is the stepwise description of net revenue calculation.

Gross revenue = Savings from landfill diversion + Carbon credits + Revenue from equivalent fuel saving + Revenue from electricity generation

The net revenue value was obtained by subtracting operational, and maintenance (O&M) costs from the gross revenue.

Net revenue = Gross revenue – Operation & maintenance cost for electricity production

The O&M costs for waste transportation, rendering facility, and transesterification unit were considered 40% of the revenue generated from electricity production. The overall projected cash flow and revenue generation from the sustainable waste management of used cooking oil and slaughterhouse waste are given in Table 4.

**Table 4:** Detailed economic analysis of esterification implementation in Makkah city.

years	Slaughterhouse waste + UCO (thousand tons)	Saving from Landfill diversion ^a^ (x 10^6^ SAR)/yr	Revenue from Carbon Credits ^b^ (x 10^6^ SAR)/yr	Revenue from Electricity generation ^c^ (x 10^6^ SAR)/yr	Revenue from oil and gas saving (x 10^6^ SAR)/yr	Gross revenue from esterification technology ^d^ (x 10^6^ SAR)/yr	Net revenue from esterification ^e^ (x 10^6^ SAR)/yr
2014	451.29	255.88	46.16	272.73	145.54	720.31	611.22
2020	525.51	297.96	53.75	317.56	169.47	838.74	711.72
2026	601.16	340.86	61.48	363.38	193.92	959.64	814.29
2032	680.65	385.93	69.61	411.40	219.54	1086.48	921.92
2038	763.41	432.85	78.08	461.40	246.23	1218.55	1034.00
2044	849.95	481.92	86.93	513.68	274.13	1356.66	1151.18
2050	940.85	533.46	96.23	568.59	303.43	1501.70	1274.27

^a^ 567 SAR/ton gate fee

^b^ at a cost of US $23.20/tonne of CO_2_. (US$ 1 = 3.750 SAR)

^c^ at SAR 0.32/kWh.

^d^ gross revenue = saving from landfill diversion + carbon credits + electricity generation + oil and gas savings

^e^ net revenue = Gross revenue–Operating & maintenance cost of esterification technology.

## 3. Results and discussion

### 3.1 Projections of pilgrims, MSW and process products

**Fig 3:**
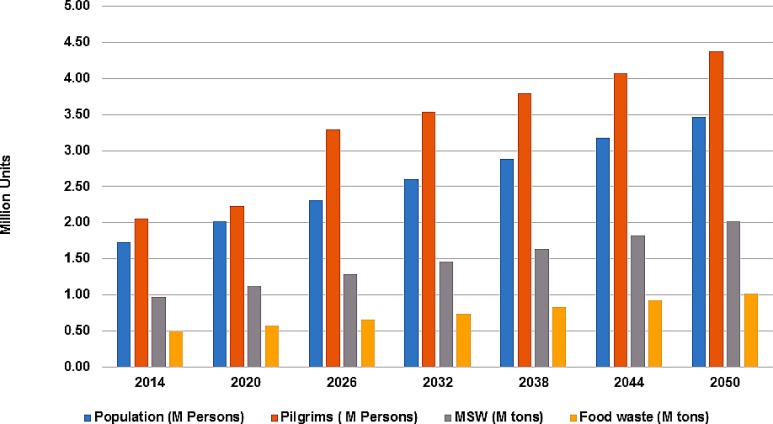
Projection of population, pilgrims, MSW and food waste fraction (million tons) in the city of Makkah from 2014 till 2050.

The population of Makkah city is projected to be around 3.5 million people with the current growth rate, while 4.4 million pilgrims will visit KSA in 2050 (Fig 3). The cumulative amount of MSW generated by the local population as well as Pilgrims will reach 2.02 million tons having 1.02 million tons of food waste stream in the year 2050 (Fig 3). The calculated amount of fat/oil fraction also showed an increasing trend (Table 1), considering its current rate that is 13.03% of the food waste fraction of MSW. The calculated amount of fat/oil fraction was 64 thousand tons in 2014, which will reach up to 133 thousand tons in 2050.

The theoretical amount of biodiesel production also changed from 63 thousand tons/yr to 130 thousand tons/yr, using 98% production yield of biodiesel with reference to input material. Similarly, the amount of glycerol production as by-product changes from 6.4 thousand tons/yr to 13.3 thousand tons/yr (Table 2). The projected amount of rendering material also increases from 451 thousand ton to 939 thousand ton, from 2014 to 2050 respectively. Similarly, MBM production potential is dependent on the rendering material processing and increases from 117 thousand tons in 2014 to 244 thousand tons in 2050 (Table 2).

### 3.2 Electricity potential of biodiesel, glycerol, and MBM

According to the base year of 2014, around 62.53 thousand tons of biodiesel could have been produced from all the fat contents of food waste generated in Makkah city (Table 2). Similarly, 6.38 thousand tons of glycerol could have been produced as a VAP and sold to the market to generate direct revenue. The HHV values of both biodiesel and glycerol were used to estimate electricity potential using 35% electricity generation efficiency [27]. The estimated potential of electricity production from biodiesel was 244.19 GWh, while 11.79 GWh electricity could have been produced from glycerol burning in the year 2014. The projected electricity generation potential per year from biodiesel and glycerol will reach up to 509.07 and 24.57 GWh in 2050 respectively, as shown in Table 2.

The MBM obtained from rendering facility is a protein rich material and used as fodder in livestock industry [16]. It can be sold directly to the market to generate direct revenue or used for energy production by burning. The annual electricity production potential from MBM has been calculated 596.30 GWh from 117 thousand tons during 2014, which will reach up to 1243.19 GWh from 244 thousand tons of MBM in 2050. It has been estimated that the overall electricity production potential from alternate resource was 852 GWh in 2014, which is expected to reach 1776.83 GWh in 2050 (Table 2).

### 3.3 Economic and environmental benefits

The transesterification is a well-known and well-established process at an industrial scale for producing biodiesel and glycerol using UCO and tallows as feedstock. Therefore, the development of this technology in Makkah city will not only solve MSW problems but also generate significant amounts of revenue. The detailed economic analysis of developing transesterification technology in Makkah city, including revenue generation from landfill diversion of waste and electricity generation and savings from carbon credits and equivalent amounts of oil and gas, from 2014 till 2050 is given in Table 4.

The diversion of materials related to fat fraction will save gate fee for sending waste to the landfills. The estimated value of gate fee savings per year could have been 256 million SAR in 2014, which is projected to reach 533 million SAR in 2050. It has been estimated that 0.047 ton CH_4_ per ton of MSW is generated in the landfill. In 2014, CH_4_ emission potential was 21405 ton, which is expected to reach 44626 ton in 2050. Since the landfill sites are not designed to collect this GHG in KSA. Therefore such massive amounts of GHG and other toxic gases are released into the atmosphere causing severe environmental and public health issues, including leachate, waterborne pollutants, and soil and water contamination along with and mosquito disease vectors [7, 11].

There is an immense potential for revenue generation by implementing carbon credits system in KSA. It has been estimated that revenue of 96 million SAR can be generated through carbon crediting in 2050. Furthermore, the generation of revenue from biodiesel and by-products was calculated based on their relevant electricity generation potential. The expected revenue from electricity generation is 273 million SAR in 2014, which is projected to reach up to 569 million SAR by 2050 (Table 4).

The estimated amount of equivalent fuel savings by alternate energy production is given in Table 5. The potential fuel saving could have been 0.83 million bbl. of oil and 3.85 million Mcf of natural gas used for energy provision in 2014. This fuel saving is expected to increase in the coming years with projected fuel saving values of 1.72 million bbl. crude oil and 8.02 Mcf of natural gas in 2050. The calculated revenue potential by selling this oil and gas share could have been 145.54 million SAR in 2014. It is expected that this fuel saving can incorporate a revenue of 303.43 million SAR in 2050 to the national economy, by exporting equivalent amount of saved fuel at current low prices (Table 5).

**Table 5:** Potential revenue in terms of oil & gas savings used for electricity production.

Years	Energy potential (GWh/yr)	Oil saving (bbl 10^6^)/yr	Revenue from oil saving (SAR 10^6^)*/yr	Natural gas saving (Mcf 10^6^)/yr	Revenue from gas saving (SAR 10^6^)**/yr	Total Savings from oil & gas saving (SAR 10^6^)/yr
2014	852.28	0.83	118.85	3.85	26.70	145.54
2020	992.38	0.96	138.39	4.48	31.08	169.47
2026	1135.55	1.10	158.35	5.13	35.57	193.92
2032	1285.61	1.25	179.28	5.80	40.27	219.54
2038	1441.86	1.40	201.06	6.51	45.16	246.23
2044	1605.24	1.56	223.85	7.25	50.28	274.13
2050	1776.83	1.72	247.77	8.02	55.65	303.43

*based on oil price: US$38.33/bbl brent crude oil [35]

**natural gas: US$1.8/MMBtu or US$1.85/Mcf [35]

The gross revenue is the sum of overall revenue generation potential from landfill diversion, carbon credits, equivalent oil and gas savings and electricity production potential. Similarly, estimated net revenue was obtained by subtracting 40% of revenue generated by electricity production, as operational and maintenance cost, from the gross revenue. The calculated potential of net revenue generation using the sustainable biodiesel production using transesterification technology was 611.22 million SAR in 2014, which is expected to reach 1274.27 million SAR in 2050 (Table 4).

### 3.4 Applications of biodiesel in Makkah city

Biodiesel has similar properties to that of mineral diesel and can be used as an alternative fuel to diesel or as a blend of mineral diesel and biodiesel in the diesel engines. Due to its eco-friendly nature and non-toxic behavior, it has certain advantages over its fossil-based competitor. The literature review reveals that it has lower emissions of particulate materials, carbon monoxide (CO) and total hydrocarbons than diesel oil [36]. It can be used as a transport fuel for diesel-fueled vehicles and heavy machinery used in the construction industry. It will significantly reduce environmental pollution coming from the combustion of diesel fuel in the form of exhaust gases [37]. The decrease in environmental pollution will lead to the cleaner atmosphere and ultimately better-living conditions for the residents. Likewise, lower emissions of CO_2_ from biodiesel consumption will help to mitigate global warming impacts. Furthermore, biodiesel is much safer to handle and almost 4 times more biodegradable by the water and soil bacterial than mineral oil [38]. With all these benefits, its production promotes material recycling and saves land, generate revenue and produce more jobs for the local population. Therefore, implementation of this technology will not only manage the fat fractions of MSW of Makkah city but also add the significant value of revenue to the national economy every year.

### 3.5 Future work

The selected transesterification technology has shown the potential for sustainable management of fat fraction of MSW arising from UCOs and slaughterhouse waste in Makkah. It will not only significantly reduce landfill and related environmental problems for Makkah but also provide an alternate energy source in the form of biodiesel and VAP such as glycerol and MBM production. Despite all the advantages of transesterification process discussed above, there are potential challenges such as free fatty acids, product purification, lower biodiesel oxidation stability, catalyst recovery and difficulties in low-temperature operations that have to be considered and tackled to achieve optimum biodiesel yield and quality [39, 40]. In order to overcome these challenges, more detailed experimental studies are required utilizing indigenous raw materials and other resources. In the next phase, more detailed experimental studies will be carried out to produce high quality biodiesel from all local waste sources, considering all the important process parameters such as reaction temperature, time, molar ratios, moisture contents in raw materials, use of various catalysts (including cheap local naturally occurring zeolite catalysts [41, 42] that significantly affect the yield and quality of biodiesel [42, 43]. These detailed experimental studies at laboratories scale will also help greatly to design and develop the biodiesel production technologies at large industrial scale.

However, the decision to implement this technology needs further in-depth technical with gathering real-time data about annual slaughtering activity and the amount of waste generation and its management, economic, social and environmental investigations using LCA and LCC methodologies [44, 45]. These studies will help to find out the hotspots and compare alternate technologies to select the best-suited option. The social norms of the local culture, human behaviour, awareness and acceptance of the modern solutions are also critical to be considered before taking the decision to implement this technology.

## 4. Conclusions

The implementation of the transesterification technology in the city of Makkah has been studied. The preliminary results revealed that processing of fat fractions from MSW and rendering material from slaughterhouses not only reduce the operational and environmental burden on the land resources but also save and generate a significant amount of economic benefits. It will process about 940 thousand ton UCOs and fat related fractions from slaughterhouse waste of MSW in 2050, producing 130 thousand ton biodiesel, 13 thousand ton glycerol and 244 thousand ton of MBM as biofuel and VAP. This processing of the waste material will save, 533 million SAR as gate fee saving from waste diversion from landfill, 96 million SAR from carbon crediting by saving CH_4_ emissions, 569 million SAR from electricity generation from biofuel and VAPs and 303 million SAR from oil and gas conservation. Overall, the implementation of this technology had the potential to add net revenue of 611 million SAR in 2014 which will increase gradually and reach up to 1274 million SAR in 2050. Although preliminary results seem very promising, further in-depth studies including local conditions, culture, and socio-economic norms are highly recommended for sustainable management of Makkah’s MSW.

## Abbreviations

AD: Anaerobic Digestion
Bbl: Barrel
CH_4_: Methane
CH_4_OH: Methanol
DOC: Degradable Organic Carbon
DOCF: Dissimilated Organic Fraction
FFAs: Free Fatty Acids
GHG: Greenhouse Gas
HHV: Higher Heating Value
IPCC: Intergovernmental Panel on Climate Change GWP
KOH: Potassium Hydroxide
KSA: Kingdom of Saudi Arabia
LCA: Life Cycle Assessment
LCC: Life Cycle Costing
LFG: Land Fill Gas
MBM: Meat and Bone Meal
MCF: Methane Correction Factor
MRF: Material Recovery Facility
MMcf: Million Mcf
MSW: Municipal Solid Waste
MSWT: Municipal Solid Waste Total
MSWF: MSW Fraction
O&M: Operational and Maintenance
SAR: Saudi Arabian Riyal
TME: Tallow Methyl Ester
UCOs: Used Cooking Oils
VAP: Value-Added Product
WTE: Waste-to-Energy
